# Multiscale Models for Fibril Formation: Rare Events Methods, Microkinetic Models, and Population Balances

**DOI:** 10.3390/life11060570

**Published:** 2021-06-17

**Authors:** Armin Shayesteh Zadeh, Baron Peters

**Affiliations:** 1 Chemical and Biomolecular Engineering, University of Illinois at Urbana-Champaign, Urbana, IL 61801, USA; armin2@illinois.edu; 2 Department of Chemistry, University of Illinois at Urbana-Champaign, Urbana, IL 61801, USA

**Keywords:** amyloid fibril growth, rare events, coarse-grained MD, population balance models

## Abstract

Amyloid fibrils are thought to grow by a two-step dock-lock mechanism. However, previous simulations of fibril formation (i) overlook the bi-molecular nature of the docking step and obtain rates with first-order units, or (ii) superimpose the docked and locked states when computing the potential of mean force for association and thereby muddle the docking and locking steps. Here, we developed a simple microkinetic model with separate locking and docking steps and with the appropriate concentration dependences for each step. We constructed a simple model comprised of chiral dumbbells that retains qualitative aspects of fibril formation. We used rare events methods to predict separate docking and locking rate constants for the model. The rate constants were embedded in the microkinetic model, with the microkinetic model embedded in a population balance model for “bottom-up” multiscale fibril growth rate predictions. These were compared to “top-down” results using simulation data with the same model and multiscale framework to obtain maximum likelihood estimates of the separate lock and dock rate constants. We used the same procedures to extract separate docking and locking rate constants from experimental fibril growth data. Our multiscale strategy, embedding rate theories, and kinetic models in conservation laws should help to extract docking and locking rate constants from experimental data or long molecular simulations with correct units and without compromising the molecular description.

## 1. Introduction

Proteins and peptides interact with water and with each other via numerous hydrophobic residues, charged residues, and hydrogen bond donors and acceptors [1,2,3]. Their diverse functional groups allow them to engage in a huge variety of intramolecular folds and intermolecular associations. The enormous number of conformational states and intramolecular interactions make the protein folding problem extremely challenging [4,5,6]. Likewise, protein–protein complex formation and peptide fibril assembly are complicated by the enormous number of intra- and intermolecular interactions.

Interest in protein folding drove tremendous efforts to develop accurate force fields [7,8,9,10], efficient molecular dynamics codes [10,11,12], advanced sampling methods [13,14,15,16,17], and data analysis tools [18,19,20,21]. Many of the MD codes and force fields developed for performing and analyzing protein folding simulations are directly useful for studies of fibril growth and other biomolecular self-assembly processes [22,23]. However, the intramolecular conformational transitions in folding and the intermolecular association steps in fibril growth also require different advanced sampling and data analysis tools [22,24,25,26].

The mechanism by which protein fibrils form has been studied extensively [27,28,29,30,31,32]. At a coarse level of detail, fibril formation begins with one or two-step [27,28] versions of one-dimensional nucleation [33,34,35,36] followed by non-equilibrium growth. Processes such as secondary nucleation, i.e., breakage of fibrils to create new nuclei, and merging of fibrils can also contribute to the fibril formation kinetics [27]. The growth mechanism and kinetics are the primary focus of this paper. Growth is thought to proceed by the addition of monomers in a two-step dock-lock mechanism [37,38,39]. The monomer first “docks” on the end of the fibril and forms initial interactions. It then “locks” into the morphology of the stable fibril structure while expelling any intervening water molecules. A single peptide can explore multiple docked states on a preformed fibril before it finally locks into the fibril’s native structure. As such, the energy surface associated with these systems is rugged, with numerous local minima. Moreover, many of the docked states are off-path and meta-stable complexes [40]. These aspects of the docked state(s) make simulation of fibril growth extremely difficult.

**Figure 1:**
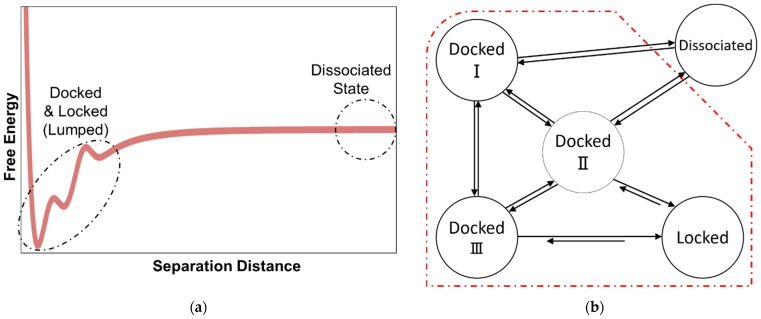
(**a**) Viewing the fibril formation process as an association problem obscures the kinetically important dock-to-lock transitions. (**b**) Markov state modeling and other approaches that begin from peptide-fibril-associated (docked) states neglect the concentration dependence of the docking step and its contribution to the fibril growth process.

Past simulations of fibril growth can be grouped into two main categories: (1) studies that compute the association constant for the combined docked and locked states [41,42,43,44,45], and (2) studies that focus on the time required to lock from the docked states, e.g., with Markov state models [46,47,48,49,50,51,52,53,54]. However, the docking and locking steps, with different concentration dependences, are rarely considered together as required for an accurate prediction of the overall growth rate. The schematics in Figure 1 depict these two incomplete modeling strategies.

Recently, Plattner et al. used a hidden Markov modeling approach coupled with the Northrup, Allison, McCammon (NAM) diffusion framework [55] to describe the association of a Barnase and Barstar complex [56]. Their atomistic simulations give rate constants with correct units: events/time/concentration for association and events/time for transitions between bound states.

Fawzi et al. studied the critical nucleus size and the elongation mechanism of two different quaternary forms of Aβ (1–40) [57]. Using a coarse-grained model of Aβ (1–40), they computed the fraction of trajectories that docked at the fibril end when started at a specific distance from the fibril. This is a key step in the NAM framework [55], but they did not complete the calculations to obtain docking and locking rate constants.

**Figure 2:**
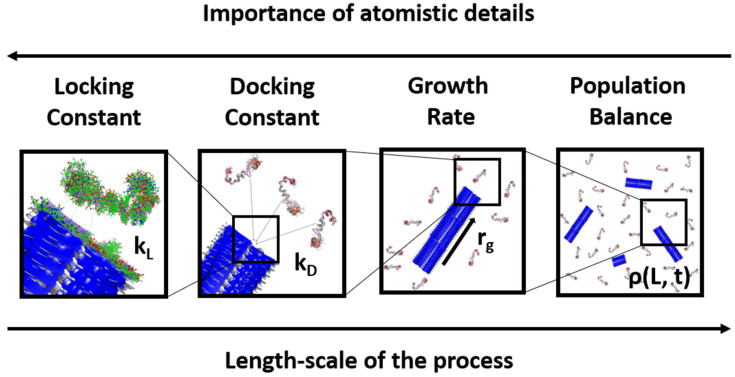
The strategy in this work exploited the multiscale nature of the fibril growth process. As we went up in length scales, the importance of treating the problem with atomistic details decreased. The protein structures depicted here are from PDB entries of Aβ−42 monomer and fibril and are for illustration purposes only.

In this article, we treated each step in the lock-dock mechanism separately, using a microkinetic model to combine both steps into an overall fibril growth rate expression. The rate expression allowed us to extract rate constants from previously obtained experimental data. Using a simple fibril assembly model, we also parametrized the microkinetic model with docking and locking rates from separate rare events calculations and compared predictions to direct simulations of fibril growth. Finally, we integrated the growth rate expression in a population balance model to describe an ensemble of growing fibrils. This multiscale treatment (Figure 2) of the growth process allowed us to directly use simulation or experimental data of fibril length vs. time to extract the rate constants for the proposed growth rate model. We demonstrate this in the last section by using our model together with experimental and simulation growth data for Aβ (1–42).

## 2. Docking and Locking Constants

For bimolecular reactions between spheres or charged spheres in solution, one can obtain rate constants from the steady-state Smoluchowski [58] or Debye [59] theories. For irregular molecules, one can monitor many reactive Brownian dynamics (BD) trajectories to calculate the rate constants. However, this is in practice impossible in an infinite domain because it requires us to simulate a huge box and wait for long times as the reactants diffuse from large distances to react (which may never happen). An alternative route to the docking rate constant $k_{D}$ is that developed by Northrup, Allison, and McCammon (NAM) [55]. NAM connected the probabilities calculated from finite domain BD trajectories to the corresponding probabilities in the infinite domain. In their method, they place the binding site of one molecule at the origin. A second molecule is placed on the surface of a sphere of radius $b_{1}$ from the origin. The distance $b_{1}$ is selected so that the two molecules interact only through an isotropic coulomb potential U(r) for separation $r>b_{1}$. A second sphere with radius $b_{2}>b_{1}$ is also centered at the origin as a cutoff distance for terminating Brownian trajectories that wander too far from the target. Multiple trajectories are then initiated from distance $b_{1}$ to estimate the probability that the second molecule docks at the fibril end (origin) before reaching the outer sphere at radius $b_{2}$

(1) $$p=\frac{\mathrm{trajectories}\;\mathrm{that}\;\mathrm{reach}\;\mathrm{docked}\;\mathrm{state}\;\mathrm{before}\;b_{2}}{\mathrm{total}\;\mathrm{no}.\;\mathrm{trajectories}}.$$

**Figure 3:**
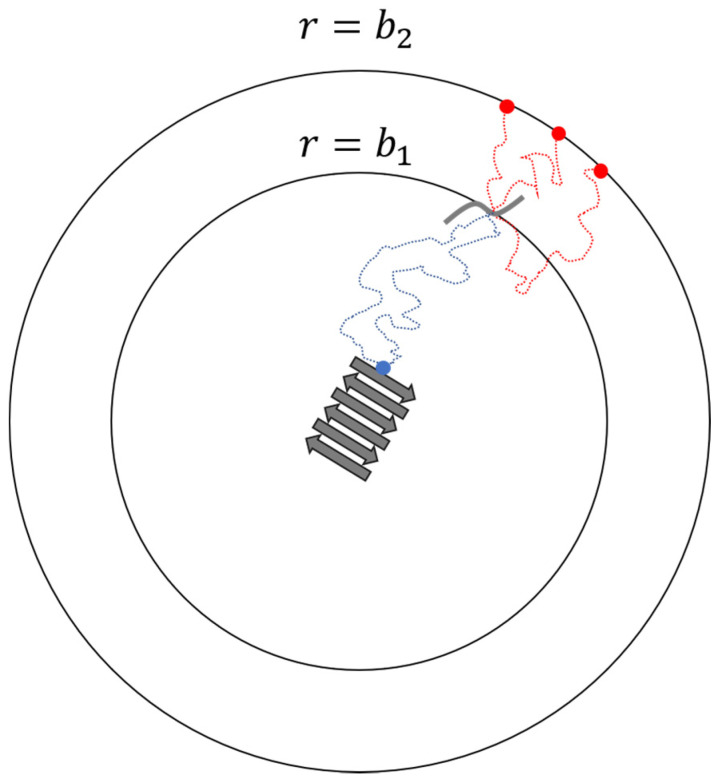
Schematics of the NAM procedure for calculating reaction rate constant of a diffusion-controlled step.

Figure 3 shows the set-up for the BD trajectories in the NAM method. Note that p is smaller than probability $p_{\infty }$ that the second molecule will dock rather than escape by diffusion to an infinite distance from the fibril end. NAM showed that

(2) $$p_{\infty }=\frac{p}{\left(1-\left(1-p\right)\Omega \right)}.$$

where Ω is the probability that a particle at $r=b_{2}$ will eventually return to $r=b_{1}$. This is obtained analytically from the Smoluchowski equation [58] as

(3) $$\Omega ={\left(\int_{b_{1}}^{\infty }dr\left[\frac{\exp\left[\beta U(r)\right]}{4\pi r^{2}D(r)}\right]\right)}^{-1}\left(\int_{b_{2}}^{\infty }dr\left[\frac{\exp\left[\beta U(r)\right]}{4\pi r^{2}D(r)}\right]\right).$$

When the potential of mean forces U(r) is zero and D is constant outside sphere with radius $b_{1}$, Equation (3) simplifies to $\Omega =b_{2}/b_{1}$. The overall rate constant can be defined as the rate of diffusion to the surface of a sphere with radius $b_{1}$ (Smoluchowski’s $4\pi D_{0}b_{1}$) [58] multiplied by the probability of reacting rather than diffusing to infinite separation, starting from separation $b_{1}$($p_{\infty }$) [55]:

(4) $$k_{d}=4\pi D_{0}b_{1}\frac{p}{\left(1-\left(1-p\right)\Omega \right)}.$$

Here, $D_{0}$ is the diffusion constant for relative motion of the particles, equal to the sum of their self-diffusion constants when no forces or hydrodynamic interactions exist for $r>b_{1}$ [55].

For complex systems where p might be extremely small, e.g., because of large peptides that interact with the fibril over long distances and therefore require a large value of $b_{1}$. These systems may require more sophisticated sampling methods such as transition interface sampling (TIS) [60] or forward flux sampling (FFS) [61] to estimate the committor p.

In atomistic simulations, the dock to lock transition is extremely complicated. Much like the protein folding problem, there are many docked states and only one (or perhaps two) locked states. Advanced sampling techniques for rare events are useful in studying the complicated dock-to-lock transition but most of these techniques require reaction coordinates that can describe the transition well. Finding a suitable reaction coordinate can be difficult [62], but there have been successful examples using native contact maps [47,48,50].

In this work, we used umbrella sampling and Langer’s method [63] to study the dock to lock transition. Starting from a set of reaction coordinates q and the free energy surface (FES) from umbrella sampling, we could expand the FES around the saddle point ($q^{‡}$) as

(5) $$F(q^{‡}+\Delta q)\approx F(q^{‡})+\frac{1}{2}\Delta q^{†}A\Delta q.$$

Matrix A should have n−1 positive eigenvalues and a single negative eigenvalue where n is the number of dimensions in q. The same free energy expansion can be used for the reactant basin

(6) $$F(q_{0}+\Delta q)\approx F(q_{0})+\frac{1}{2}\Delta q^{†}A_{0}\Delta q,$$

where $q_{0}$ is the coordinate of the bottom of the well of the reactant basin. The diffusion tensor at the saddle point is also needed for Langer’s theory

(7) $$2D_{ij}t={\langle \delta q_{i}(t)\delta q_{j}(t)\rangle }_{‡}.$$

Here, $\delta q_{i}\left(t\right)=q_{i}\left(t\right)-{\langle q_{i}\left(t\right)\rangle }_{‡}$ and the double dagger sign indicates that the average is calculated using trajectories started at the saddle point. Having calculated $D_{ij}$, we could obtain the locking rate constant from

(8) $$k_{L}=\frac{1}{2\pi }{\left(\frac{\det A_{0}}{\left|\det A\right|}\right)}^{1/2}\lambda_{+}\exp\left(-\beta \left(F(q^{‡})-F(q_{0})\right)\right),$$

where $-\lambda_{+}$ is the single negative eigenvalue of the matrix DA,

(9) $$DAu=-\lambda_{+}u.$$

## 3. Microkinetic Model

Although many studies refer to one step as fast [39,64], the docking and locking processes happen at the same net rate when fibril is growing at a steady state. The forward and backward docking and locking rates also depend differently on the monomer concentration, and thus $k_{D}$ and $k_{L}$ have different units. The docking process is a diffusion-controlled, bi-molecular association process, and therefore $k_{D}$ has units of L/mol/s [44,65]. The locking process, however, is a conformational rearrangement with a first-order rate constant similar to a protein folding process, with units of events/s [47].

An overall rate law for the dock-lock growth mechanism can be developed using microkinetic modeling techniques that are familiar in catalysis [66]. For example, Massi and Straub developed a microkinetic model for the elongation rate of a fibril [65]. They included two different states for both the fibril and the monomer and derived a rate expression with six different rate constants. Massi and Straub’s work is too elaborate to be fitted with most datasets, but they extracted each constant using multiple fitting techniques and data from multiple experimental methods.

**Figure 4:**
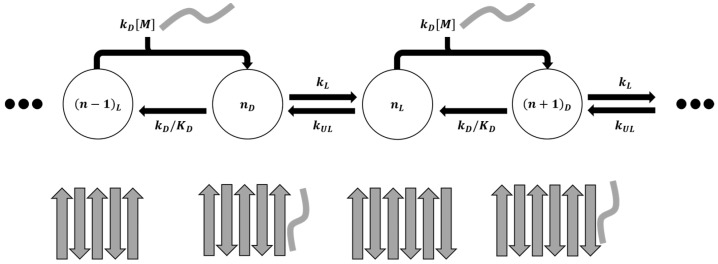
Schematic showing overall dock-lock growth mechanism as a cycle of elementary steps.

Here, we try to simplify the growth rate expression by proposing a model where the fibril end is always in either a docked state or a locked state. The underlying assumption is that the docked states of the system inter-convert on a faster timescale than the locking process and the overall growth process, and therefore all the docked states can be lumped into one. This means that the system has no on- or off-path meta-stable states other than the docked state mentioned above. We further assumed that the monomers can only bind to a locked end. Figure 4 shows the overall schematic of the growth process.

By using pseudo-steady state approximation and computing the net flux between any docked and locked state, we obtained the net growth rate as [66]

(10) $$r_{\mathrm{growth}}=\frac{k_{L}k_{D}[M]-k_{D}K_{D}^{-1}k_{UL}}{k_{L}+k_{D}K_{D}^{-1}+k_{UL}+k_{D}[M]}.$$

Here, $k_{D}$ is the docking rate constant, $k_{L}$ is the locking rate constant, [M] is the monomer(peptide) concentration, $k_{UL}$ is the reverse rate for the locking step, and $K_{D}$ is the equilibrium constant for the docking step.

## 4. Population Balance Model

Here, we assume that fibrils grow irreversibly, e.g., because the monomers do not unlock once they are locked into a fibril ($k_{UL}=0$) or because the docking equilibrium constant $K_{D}$ is large. If we take the case where $k_{UL}=0$, then the growth rate equation will be

(11) $$r_{g}=\frac{k_{L}k_{D}[M]}{k_{L}+k_{D}K_{D}^{-1}+k_{D}[M]}$$

and the following master equation describes the fibril population

(12) $$\frac{d\rho (L,t)}{dt}=-r_{g}([M])\rho (L,t)+r_{g}([M])\rho (L-1,t).$$

The equation for dρ/dt would also contain ρ(L+1,t) terms if we considered fibril dissolution. Expanding ρ(L−1,t) around L and keeping terms up to the second order gives

(13) $$\rho (L-1,t)=\rho (L,t)-\frac{\partial \rho (L,t)}{\partial L}+\frac{1}{2}\frac{\partial^{2}\rho (L,t)}{\partial L^{2}}.$$

Putting Equation (13) back into Equation (12) gives

(14) $$\frac{\partial \rho (L,t)}{\partial t}+r_{g}([M])\frac{\partial \rho (L,t)}{\partial L}=\frac{r_{g}([M])}{2}\frac{\partial^{2}\rho (L,t)}{\partial L^{2}}.$$

This is now a Fokker–Planck equation for variable L. We note that Equation (14) only describes growth. Nucleation, aggregation, and breakage of fibrils can be included in more elaborate PBEs [67], but we do not attempt that here. Moreover, note that there are no nucleation or fragmentation events in the PDE, and thus the total number of fibrils is constant,

(15) $$\rho_{\mathrm{tot}}=\int_{0}^{\infty }\rho (L,t)dL\;\forall t.$$

In a finite closed system with a fixed number of monomers, the concentration of free dissolved monomers [M] decreases as they attach to the fibrils:

(16) $$\frac{d[M]}{dt}=-r_{g}([M])\int_{0}^{\infty }\rho (L,t)dL.$$

Equations (14) and (16) describe the case where the fibril grows from only one end. In cases where the fibril can grow from both ends, the coefficients for length derivatives in Equation (14) and the right side of Equation (16) should be multiplied by a factor of 2.

The monomer concentration might be held constant at [*M*] = [*M*]_0_ throughout the experiment, e.g., by a continuous flow of solution over immobilized fibrils. In this case, Equation (14) can be simplified by the substitution

(17) $$dt=r_{g}({\left[M\right]}_{0})dt$$

to give

(18) $$\frac{\partial x(L,t)}{\partial t}+\frac{\partial x(L,t)}{\partial L}=\frac{1}{2}\frac{\partial^{2}x(L,t)}{\partial L^{2}},$$

where $x(L,t)=\rho (L,t)/\rho_{\mathrm{tot}}$. Full derivation steps are available in the accompanying Supplementary Information. The solution to Equation (18) with the initial distribution

(19) $$x(L,0)=x_{0}(L)$$

Is

(20) $$x(L,t)=\int_{0}^{\infty }dL_{0}g(L,t|L_{0})x_{0}(L_{0}).$$

Here, $g(L,t|L_{0})$ is the Green’s function given by

(21) $$g(L,t|L_{0})=\frac{1}{\sqrt{2\pi t}}\exp\left(-\frac{{\left(L-L_{0}-t\right)}^{2}}{2t}\right).$$

**Figure 5:**
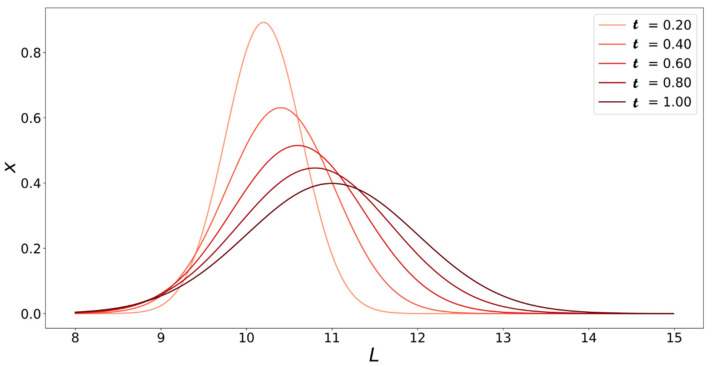
Solutions to Equation (20) plotted at different values of t as a function of fibril length (L, number of monomers). Initial fibril size distribution is $x_{0}(L)=\delta (L-10)$.

Figure 5 shows the solution to the population balance model at various dimensionless times t vs. fibril length L.

When the system has a fixed total number of peptides so that growth depletes monomer concentration, Equation (16) can be written as

(22) $$\frac{1+\alpha m}{\left(1+\alpha \right)m}dm=-\varepsilon dt,$$

where $m\equiv [M](t)/{\left[M\right]}_{0}$, $\alpha \equiv k_{D}k_{L}^{-1}{\left[M\right]}_{0}/(1+k_{D}K_{D}^{-1}k_{L}^{-1})$, $\varepsilon \equiv \rho_{\mathrm{tot}}/{\left[M\right]}_{0}$, and $\rho_{\mathrm{tot}}$ is defined as in Equation (15). The solution to Equation (22) is

(23) $$m(t)=\frac{1}{\alpha }W\left[\alpha e^{\alpha }e^{-\varepsilon t}e^{-\alpha \varepsilon t}\right].$$

In Equation (23), W(z) is the Lambert W function, which is the solution w to $z=we^{w}$. Using Equation (23), we can rewrite the population balance model in Equation (14) for a system with a fixed number of peptides. Division through by $r_{g}({\left[M\right]}_{0})$ and $\rho_{\mathrm{tot}}$ gives

(24) $$\frac{\partial x(L,t)}{\partial t}+\frac{r_{g}([M])}{r_{g}({\left[M\right]}_{0})}\frac{\partial x(L,t)}{\partial L}=\frac{1}{2}\frac{r_{g}([M])}{r_{g}({\left[M\right]}_{0})}\frac{\partial^{2}x(L,t)}{\partial L^{2}}.$$

Dividing Equation (24) by $r_{g}([M])/r_{g}({\left[M\right]}_{0})$ now gives

(25) $$\frac{\partial x(L,\tau )}{\partial \tau (t)}+\frac{\partial x(L,\tau )}{\partial L}=\frac{1}{2}\frac{\partial^{2}x(L,\tau )}{\partial L^{2}},$$

where $d\tau (t)=r_{g}([M])/r_{g}({\left[M\right]}_{0})dt=(1+\alpha )m/(1+\alpha m)dt$. The initial distribution for Equation (25) is

(26) $$x(L,0)=x_{0}(L).$$

The solution to Equation (25) will have the same format as Equation (18), i.e.,

(27) $$x(L,\tau )=\int_{0}^{\infty }dL_{0}g(L,\tau |L_{0})x_{0}(L_{0})$$

with τ defined as

(28) $$\tau =\int_{0}^{t}\frac{m(t)\left(1+\alpha \right)}{1+\alpha m(t)}dt=\frac{1}{\varepsilon }\left[1-\frac{1}{\alpha }W\left[\alpha e^{\alpha }e^{-\left(1+\alpha \right)\varepsilon t}\right]\right].$$

**Figure 6:**
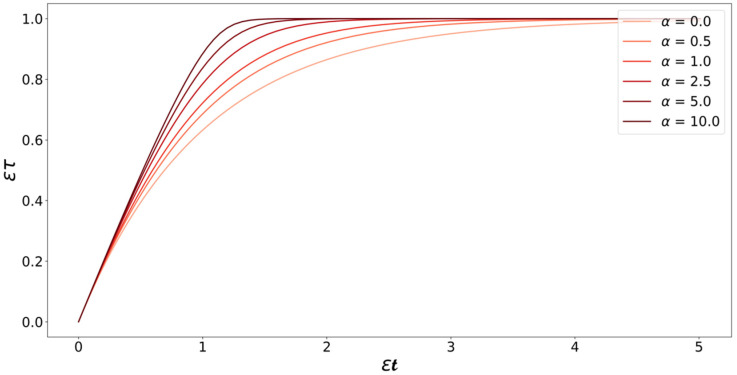
Plot of Equation (28) at different values of α. The curves start with a slope of one at low values of t, which is where the monomer concentration is still high and plateau as time passes and monomers are consumed. After the monomers are consumed, the slopes of the curves become zero, indicating that the addition of monomers to the fibrils has stopped.

Note that all the variables and kinetic parameters of the system (i.e., ${\left[M\right]}_{0}$, $\rho_{\mathrm{tot}}$, $k_{D}$, $k_{L}$, and $K_{D}$) are now absorbed into α and ε. Figure 6 illustrates the relation between τ and t for different values of α. The relation between τ and t changes from equality in the constant monomer concentration case to a Lambert W function in the monomer depletion case. Equation (28) shows that the time scale of the growth process “stretches” as monomers are consumed. This is consistent with our intuition—as the monomer concentration decreases, the driving force for growth reduces, it takes longer for the monomers to find fibril ends, and the time needed for the addition of monomers to the fibril increases.

**Figure 7:**
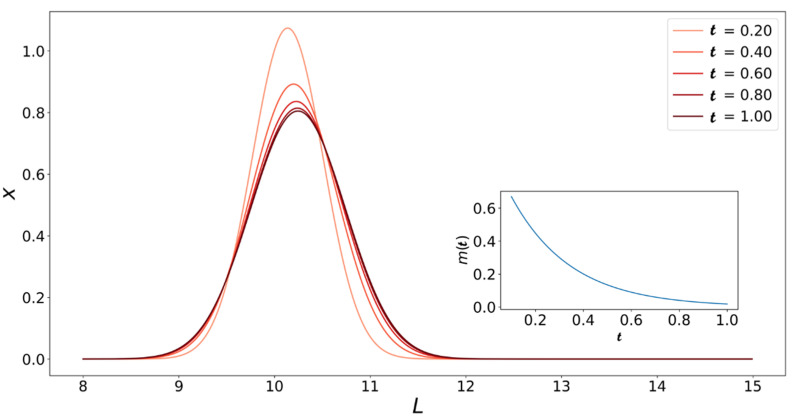
Solutions to Equation (27) with τ defined by Equation (28). Initial fibril length distribution is $x_{0}(L)=\delta (L-10)$. The same values of α and ε are used so that Figure 5 and Figure 7 are comparable. The inset shows the change in dimensionless monomer concentration m with dimensionless time (Equation (23)).

We can also use Equation (28) to create a plot like Figure 5 for the varying monomer concentration case. Figure 7 shows how the fibril populations change with t when the monomer concentration is not held constant. As expected, the population of fibrils with L=10 (initial length) grows towards longer fibrils until the system runs out of monomers. The growth process is also slowed down by the fact that the driving force for growth (i.e., monomer concentration) is constantly decreasing. This means that at the same t, the peaks in Figure 7 are higher and at lower L values compared to their counterparts in Figure 5.

The analysis laid out above allows us to use time-series data of monomer concentration and fibril length from simulation data or experimental results together with Equation (21) or Equation (27) to extract values for $k_{D}$, $K_{D}$, and $k_{L}$.

## 5. Simple Model and Simulations

**Figure 8:**
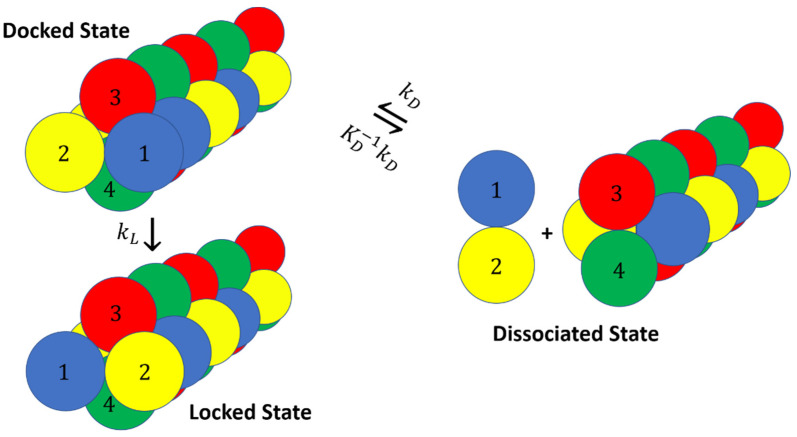
Dumbbells model. The addition of a dumbbell to the fibril happens through a two-step mechanism where the dumbbell initially docks with the wrong chirality and then locks into the correct chirality. The dumbbell and the fibril end have the same $r_{\mathrm{COM}}$ value but different q values in the docked and locked states.

To test our rate equation with simulation data, we developed a simple model system with two types of diatomic molecules that associate in “docked” or “locked” states. The docked and locked states in our model correspond to fibril ends with a specific chirality as shown in Figure 8. The interaction potential is made up of several components:

(29) $$\begin{array}{cc} U_{\mathrm{tot}} & =\sum_{\mathrm{bonds}}\frac{k_{b}}{2}{\left(r-r_{0}\right)}^{2}\\ & +\sum_{\mathrm{atoms}}4\varepsilon_{\mathrm{lj}}\left({\left(\frac{r_{\mathrm{lj}}^{m}}{2^{1/6}r}\right)}^{12}-{\left(\frac{r_{\mathrm{lj}}^{m}}{2^{1/6}r}\right)}^{6}\right)\\ & +\sum_{\mathrm{dumbbells}}4\varepsilon_{\mathrm{lj},c}\left({\left(\frac{r_{\mathrm{lj},c}^{m}}{2^{1/6}r_{\mathrm{COM}}}\right)}^{12}-{\left(\frac{r_{\mathrm{lj},c}^{m}}{2^{1/6}r_{\mathrm{COM}}}\right)}^{6}\right)\\ & +\sum_{\mathrm{atoms}}^{r\leq r_{\mathrm{WCA}}^{m}}\left[4\varepsilon_{\mathrm{WCA}}\left({\left(\frac{r_{\mathrm{WCA}}^{m}}{2^{1/6}r}\right)}^{12}-{\left(\frac{r_{\mathrm{WCA}}^{m}}{2^{1/6}r}\right)}^{6}\right)+\varepsilon_{\mathrm{WCA}}\right]\\ & +\sum_{\mathrm{dumbbells}}e_{w}\exp\left(\frac{-{\left(r_{\mathrm{COM}}-r_{0,w}\right)}^{2}}{2s_{r,w}^{2}}\right)\exp\left(\frac{-{\left(q-q_{0,w}\right)}^{2}}{2s_{q,w}^{2}}\right)\\ & +\sum_{\mathrm{dumbbells}}e_{c}\exp\left(\frac{-{\left(r_{\mathrm{COM}}-r_{0,c}\right)}^{2}}{2s_{r,c}^{2}}\right)\exp\left(\frac{-{\left(q-q_{0,c}\right)}^{2}}{2s_{q,c}^{2}}\right)\\ & +\sum_{\mathrm{dumbbells}}e_{t}\exp\left(\frac{-{\left(r_{\mathrm{COM}}-r_{0,t}\right)}^{2}}{2s_{r,t}^{2}}\right)\exp\left(\frac{-{\left(q-q_{0,t}\right)}^{2}}{2s_{q,t}^{2}}\right). \end{array}$$

**Figure 9:**
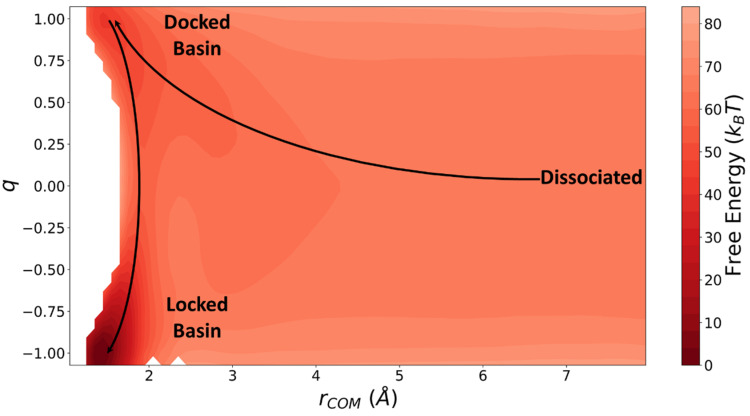
Free energy surface obtained from umbrella sampling and weighted histogram analysis [69]. The solid lines show the path of a typical reactive trajectory.

The components shown in Equation (29) are defined as follows (line by line):

1. Particles of each dumbbell are bonded together with a harmonic potential (1–2 and 3–4 interactions in Figure 8).
2. Particles of types 1 and 2 interact with particles of types 3 and 4 through a Lenard–Jones potential. This potential contributes equal stability to both the docked and locked states.
3. The centers of mass (COM) of molecules of type 1–2 and 3–4 interact through a short-ranged Lenard–Jones potential. This potential stabilizes the fibril and prevents it from dissociating. Inclusion of these LJ interactions also allows us to reduce the strength of LJ interactions between edges of the fibril and molecules in solution, which might otherwise promote branching and secondary nucleation.
4. A Weeks–Chandler–Andersen potential between the particles in the same type of molecule, i.e., between 1 and 1, 1 and 2, 2 and 2, 3 and 3, 3 and 4, and 4 and 4, prevents the fibril from forming unstructured oligomers.
5. A channel that guides incoming dumbbells to the docked state is introduced by using a combination of three 2D Gaussian functions (lines 5, 6, and 7 in Equation (29)):
  - The wall: This part of the force field prevents the dumbbells from directly locking into the fibril (vertical wall near $r_{\mathrm{COM}}=2$ in Figure 9).
  - The channel: This guides the trajectories into the docked basin after they pass the wall.
  - Locked state tilting: This Gaussian function is included to make the locked state more favorable than the docked state.

Values of the energy and geometry parameters of the interaction potential are shown in Table 1. 

The reaction coordinates that describe the dumbbells and their relative positions are as follows: $r_{\mathrm{COM}}$ is the center-of-mass to center-of-mass coordinate distance between two dumbbells, and q is a chirality coordinate that describes the structure of each dimer. These are defined for each pair of molecules using the positions of the particles 1 through 4 via the following definitions:

(30) $$\vec{u}=\frac{1}{2}({\vec{x}}_{1}+{\vec{x}}_{2})-\frac{1}{2}({\vec{x}}_{3}+{\vec{x}}_{4}),$$

(31) $$\vec{v}=({\vec{x}}_{4}-{\vec{x}}_{3})\times ({\vec{x}}_{2}-{\vec{x}}_{1}),$$

(32) $$r_{\mathrm{COM}}=\frac{\left\|u\right\|}{r_{0}},$$

(33) $$q=\frac{u.v}{r_{0}{}^{2}\left\|u\right\|}.$$

Here, $r_{0}$ is the equilibrium bond length. The chirality coordinate is defined to differentiate between the two enantiomers. Values of q range from −1 to 1, where −1 and 1 (at a known separation r) are the two possible enantiomers. The extrema of q are not exactly equal to −1 and 1, due to the vibrations of the bonds during the simulation.

All the trajectories are generated following the overdamped dynamics and using the Euler–Maruyama integrator [68]

(34) $$x(t+\Delta t)=x(t)-D\frac{\partial \beta F}{\partial x}\Delta t+\sqrt{2D\Delta t}\xi ,$$

where D is diffusivity (identical for all atoms), $\beta =1/k_{B}T$, and ξ is a Gaussian random number with zero mean and unit variance.

NAM analysis is performed following the protocol described in Section 2 by putting a fibril made of four dumbbells in the center of the simulation box and a dumbbell of type 3–4 at a given distance $b_{1}$. The trajectories will run until the distance between the dumbbells becomes either smaller than the average center-to-center distance in the docked (or locked) state or greater than the predefined cut-off distance $b_{2}$. Depending on the starting separation, 10,000 or 20,000 trajectories are launched from distance $b_{1}$= 12, 13, 14, 15, and 16 Å. The number of successful trajectories are counted, and success probability is then calculated using Equation (1). The number of trajectories that go directly to the locked state is also tracked to make sure that nearly all dumbbells initially attach in the docked state.

The free energy profile between a dumbbell and a preformed fibril of length L=6 as a function of $r_{\mathrm{COM}}$ and q is calculated using umbrella sampling. The free energy profile is then used in Langer’s theory as described in Section 2 to calculate the locking rate constant. The docking equilibrium constant ($K_{D}$) is also calculated using the free energy profile.

## 6. Results and Discussion

The docking rate was computed through the NAM method described in Section 2. Table 2 shows results for different starting separations $b_{1}$ and cutoff distances $b_{2}$.

The success probability p was calculated using Equation (1). The results presented in Table 2 show that a very low percentage of trajectories directly went to the locked state, and thus the dynamics of the model system were consistent with the assumed sequence of events in the dock-lock mechanism. The docking rates presented in the table were calculated using Equation (4). As expected, the computed value $k_{D}=(1.21\pm 0.01)\times 10^{9}$ L/mol/s, did not depend on $b_{1}$ and $b_{2}$.

The trajectories that fell directly into the locked state were omitted from the results above and were assumed to introduce negligible error in the rate calculation procedure (less than ≈6% of the trajectories).

Bottom-up rate constants were obtained using free energy surface and short trajectories with methods in Section 2. Specifically, we calculated the diffusivity tensor used in Langer’s theory using pico-second long trajectories started near the saddle point. Using Equation (7), we calculated diffusivities as $D_{rr}=1.696\times 10^{10}$ Å^2^/s, $D_{qq}=1.600\times 10^{10}$/s, and $D_{rq}=D_{qr}=7.808\times 10^{8}$Å/s. The free energy surface obtained from umbrella sampling (shown in Figure 9) was used to expand the free energy around the minimum of the docked basin and the saddle point. Finally, we used Equation (8) to calculate $k_{L}=9.852\times 10^{7}$/s. The docking equilibrium constant was also calculated from the free energy surface to be $K_{D}=8.440\times 10^{2}$ mol/L.

To demonstrate how the population balance model can be used in top-down data analysis to extract rate parameters, we filled a periodic simulation box of size (60 Å)^3^ with individual dumbbells and a preformed fibril of initial length 10 dumbbells. We prevented the nucleation of new fibrils by constantly checking for dimers, separating them, and (randomly) throwing the dumbbells back in the box. The dumbbell concentration was held constant by adding a dumbbell to the box in a random position every time the fibril grew. Results from this simulation were used with maximum likelihood estimation (MLE) to extract the rate constants from brute-force simulations. Results were compared to the values calculated from Langer’s theory.

**Figure 10:**
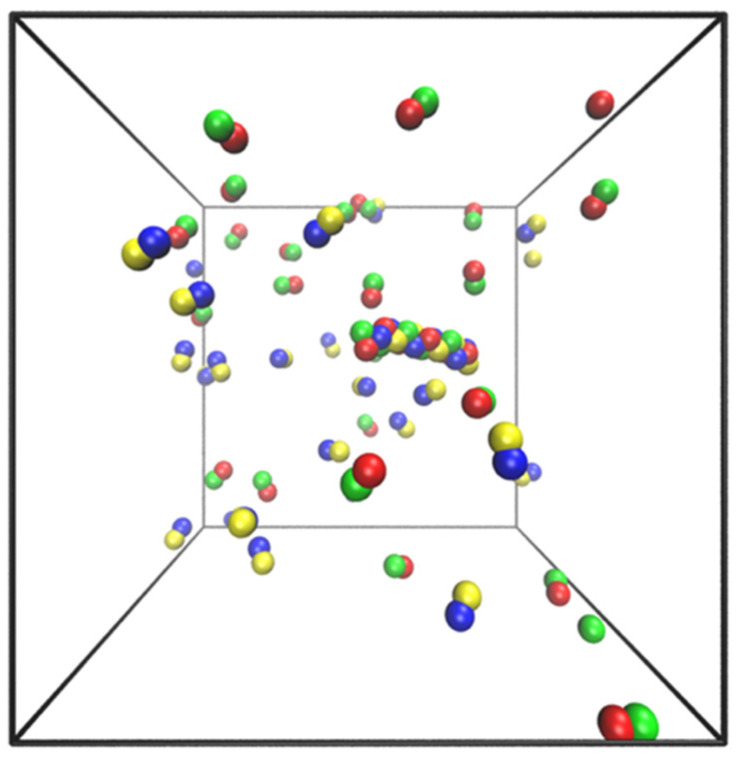
Snapshot of the simulation box with a single fibril of length 10 and 50 dumbbells visualized by VMD [70].

Top-down estimates of the same rate constants were obtained from simulations of fibril growth in 50 identical boxes at five different concentrations, each one held at fixed dumbbell concentration with seeded fibrils of length $L_{0}=6$. Figure 10 shows a snapshot of the simulation box. The data from these simulations were then used to extract rate constants with MLE. To this end, the growth rate expression in Equation (11) had to be re-casted in a form that enabled the identification of free parameters for optimization. Division through by $k_{D}$ gave

(35) $$r_{g}=\frac{k_{L}[M]}{K+[M]}$$

where $K=k_{L}/k_{D}+K_{D}{}^{-1}$. The code used for the top-down analysis is available in the Supplementary Information. Table 3 shows the results from top-down analysis and bottom-up predictions side-by-side.

There was reasonable (order-of-magnitude) agreement between the values for $k_{L}$ and K as calculated from rare events methods and MLE. The difference between the bottom-up and the top-down $k_{L}$ values could arise for two reasons. First, errors in the computed free energy surface were exponentially magnified in Langer theory for $k_{L}$. Second, the data provided to MLE for fitting was limited to ≈100 ns at five different monomer concentrations. The predictions would be more accurate with longer trajectories and a wider range of concentrations. MLE code and the contour plot showing the log-likelihood as a function of $k_{L}$ and K are available in the Supplementary Information.

To further demonstrate the top-down extraction of rate constants from MLE with our population balance model and growth rate expression, we used previously published experimental data from Young et al. [71] for the growth of Aβ (1–42). In their analysis, Young et al. used a two-step Michaelis–Menten kinetic model to describe the results. In this work, we derived our rate law for fibril growth on the basis of the two-step dock-lock mechanism and by using microkinetic model techniques. This provided a mechanistic basis for the Michaelis–Menten expression and a molecular interpretation for its fit parameters. We calculated $k_{L}=21.10$ /s and $K=2.71\times 10^{-5}$ mol/L. Young et al. reported a value of $K=(7.2\pm 2.4)\times 10^{-5}$ mol/L. Note that Young et al. invoked an additional “pause state” beyond the dock and lock states to explain intermittent plateaus in their fibril length vs. time data. They did not provide details on how they treated the pauses in their data analysis, e.g., whether they truncated or abridged the data to remove pauses in computing K. We obtained the value $K=2.71\times 10^{-5}$ mol/L by fitting their entire dataset with no adjustments. Potential differences in our analysis strategies may therefore have been responsible for the threefold difference in estimates of K. 

## 7. Conclusions

This study presented a multiscale rare events strategy for the dock-lock mechanism of fibril growth. Our strategy separately computes dock and lock rate constants with correct units and incorporates them in a microkinetic model for the growth (or dissolution) rate as a function of monomer concentration. The microkinetic model is further embedded in a population balance model to predict the evolving length distribution in an ensemble of growing fibrils. The population balance model provides a direct connection to experimental data and/or to large-scale molecular simulation data of fibril growth. We demonstrated how this framework can be used for top-down extraction of kinetic parameters from experimental data and molecular simulation data. For the simulation data, we demonstrated that the top-down analysis yielded rate constants in reasonable agreement with those from rare events calculations for the dock and lock steps. 

Apart from the multiscale framework, a central contribution of this work is a blueprint for computing second-order docking rate constants and constructing proper concentration-dependent fibril growth rate laws from molecular level rare events calculations. Although fibril growth mechanisms will vary by sequence and solvent composition, possibly introducing additional intermediates and kinetic traps, the multiscale analysis strategy here should help to connect simulations and experiments. We note that it should also be possible to obtain second-order docking rate constants from MSM results, but to our knowledge, the calculation has not been done. As outlined in Joswiak et al. [72], one must first assume that fibril–solute encounters are rare, i.e., that finding a solute in the simulation box is already a rare event. Then, Poisson statistics give the probability to find a solute in the simulation box as $V_{\mathrm{box}}[M]$, where $V_{\mathrm{box}}[M]<<1$. Now suppose the MSM approach finds a docking rate constant $k_{\mathrm{MSM}}$ with units/s. The MSM result is a conditional transition probability per time given that a solute is in the box. This means that the true rate, using conditional probability rules, is

(36) $$r_{D}=k_{\mathrm{MSM}}V_{\mathrm{box}}[M].$$

From this, we identify $k_{D}=k_{\mathrm{MSM}}V_{\mathrm{box}}$, which should make it possible to revisit many MSM predictions, convert them to second-order $k_{D}$ values, and compare them to experiments. 

## Figures and Tables

**Table 1:** Values and definitions of the interaction potential parameters.

Parameter	Value	Description
Bonded Potential		
$k_{b}$	1000 k_B_T/Å^2^	Bond strength parameter
$r_{0}$	2.0 Å	Equilibrium bond length
**Lennard–Jones and WCA Potentials**		
$\varepsilon_{\mathrm{lj}}$	3.0 k_B_T	Depth of atom–atom LJ potential well
$r_{\mathrm{lj}}^{m}$	2 Å	Atom–atom LJ equilibrium bond length
$\varepsilon_{\mathrm{lj},c}$	5.0 k_B_T	Depth of COM–COM LJ potential well
$r_{\mathrm{lj},c}^{m}$	1.4 Å	COM–COM LJ equilibrium bond length
$\varepsilon_{\mathrm{WCA}}$	15.0 k_B_T	Depth of WCA potential well
$r_{\mathrm{WCA}}^{m}$	2.8 Å	WCA equilibrium bond length
**Gaussian Potentials**		
$e_{w}$	6.0 k_B_T	Peak size of the wall
$r_{0,w}$	2.2 Å	Location of the wall peak on the $r_{\mathrm{COM}}$ axis
$s_{r,w}$	0.4 Å	SD of the wall (width in $r_{\mathrm{COM}}$)
$q_{0,w}$	−1.0	Location of the wall peak on the q axis
$s_{q,w}$	1.2	SD of the wall (width in q)
$e_{c}$	−8.0 k_B_T	Peak size of the channel
$r_{0,c}$	1.7 Å	Location of the channel peak on the $r_{\mathrm{COM}}$ axis
$s_{r,c}$	1.0 Å	SD of the channel (width in $r_{\mathrm{COM}}$)
$q_{0,c}$	0.65	Location of the channel peak on the q axis
$s_{q,c}$	0.6	SD of the channel (width in q)
$e_{t}$	−35.0 k_B_T	Peak size of the tilting
$r_{0,t}$	1.4 Å	Location of the tilting peak on the $r_{\mathrm{COM}}$ axis
$s_{r,t}$	0.4 Å	SD of the tilting (width in $r_{\mathrm{COM}}$)
$q_{0,t}$	−1.0	Location of the tilting peak on the q axis
$s_{q,t}$	0.6	SD of the tilting (width in q)

**Table 2:** Docking rate constants $k_{D}$ calculated from NAM simulations.

$\mathrm{Initial}\;\mathrm{Distance}\;b_{1}\;(Å)$	$\mathrm{Cut-Off}\;\mathrm{Distance}\;b_{2}\;(Å)$	p %	$k_{D}$ (L/mol/s)	% Directly to Locked
12	20	22	1.30 × 10^9^	5.40
13	22	16	1.11 × 10^9^	0.00
14	24	17	1.23 × 10^9^	3.21
15	26	15	1.20 × 10^9^	4.11

**Table 3:** Rate constants calculated by rare events methods (bottom-up) and by MLE (top-down).

Rate Constant	Rare Events	MLE
$k_{L}$ (/s)	9.8 × 10^7^	8.3 × 10^6^
K (mol/L)	0.08	0.07

## Supplementary Materials

The following are available online at https://www.mdpi.com/article/10.3390/life11060570/s1, Derivations of Equation (18), the code for MLE analysis, Figure S1: The contour plot of the negative log-likelihood around the minimum.
