# Supplementary material for: Multiscale Models for Fibril Formation: Rare Events Methods, Microkinetic Models, and Population Balances
Source: Life (Basel). 2021 Jun 17;11(6):570. doi: 10.3390/life11060570 (PMC8234428; doi:10.3390/life11060570)
Supplement: Supplementary file 1 [file life-11-00570-s001.zip › life-1203455-supplementary.pdf]

Supplementary Information:

Multiscale models for fibril formation: rare events methods, microkinetic models, and population balances

Armin Shayesteh Zadeh and Baron Peters

S1. Derivation of the population balance model for varying monomer concentration case (Eq (18))

The growth rate from Eq (2) can be reorganized to give

$$r_g(M) = k_D M \frac{1}{1 + k_D K_D^{-1} k_L^{-1} + k_D M k_L^{-1}}. \tag{S.1}$$

Division of Eq (S.1) by $r_g(M_0)$ gives

$$\frac{r_g(M)}{r_g(M_0)} = m\frac{1+a+c}{1+a+cm} = \frac{m(1+\alpha)}{1+\alpha m} \tag{S.2}$$

where $m \equiv \dfrac{M}{M_0}$, $a \equiv k_D K_D^{-1} k_L^{-1}$, $c \equiv k_D M_0 k_L^{-1}$, and $\alpha \equiv c/(1+a)$.

Using Eq (6) in Eq (7) gives

$$\frac{dM}{dt} = -r_g(M)\rho_{tot}. \tag{S.3}$$

Division of both sides of Eq (S.3) by $M_0 r_g(M_0)$ gives

$$\frac{dm}{d\boldsymbol{t}} = -\frac{m(1+\alpha)}{1+\alpha m}\varepsilon \tag{S.4}$$

with $\varepsilon = \rho_{tot}/M_0$ and $\boldsymbol{t}$ defined by Eq (8). Integration of Eq (S.4) gives Eq (14):

$$\int_1^m \frac{1+\alpha m}{m(1+\alpha)} dm = -\int_0^{\boldsymbol{t}} \varepsilon d\boldsymbol{t} \tag{S.5}$$

$$\rightarrow \frac{\alpha(m-1) + \log(m)}{1+\alpha} = -\varepsilon \boldsymbol{t} \tag{S.6}$$

$$\rightarrow m(\boldsymbol{t}) = \frac{1}{\alpha} W\left[\alpha e^{\alpha} e^{-\varepsilon \boldsymbol{t}} e^{-\alpha\varepsilon \boldsymbol{t}}\right]. \tag{S.7}$$

We can now go back to Eq (5) and re-write it in a dimensionless manner. Division of both side by $\rho_{tot} r_g(M_0)$ gives:

$$\frac{\partial x(L,\boldsymbol{t})}{\partial \boldsymbol{t}} + \frac{m(1+\alpha)}{1+\alpha m}\frac{\partial x(L,\boldsymbol{t})}{\partial L} = \frac{1}{2}\frac{m(1+\alpha)}{1+\alpha m}\frac{\partial^2 x(L,\boldsymbol{t})}{\partial L^2}. \tag{S.8}$$

We can now define another dimensionless time

$$d\tau = \frac{r_g(M)}{r_g(M_0)} d\boldsymbol{t} = \frac{m(1+\alpha)}{1+\alpha m} d\boldsymbol{t} \tag{S.9}$$

So Eq (S.8) simplifies to

$$\frac{\partial x(L,\tau)}{\partial \tau} + \frac{\partial x(L,\tau)}{\partial L} = \frac{1}{2}\frac{\partial^2 x(L,\tau)}{\partial L^2}. \tag{S.10}$$

Eq (S.10) looks similar to Eq (9) except that $\boldsymbol{t}$ is replaced with τ, so the solution to Eq (S.10) will look like Eq (11) with τ as the dimensionless time variable (Eq (18)).

S2. Python code for using maximum likelihood estimation on experimental data

The following is the Python code used to extract rate constants for the growth rate expression from computational or experimental fibril length vs time data.

```
import numpy as np
from scipy.optimize import minimize

def neg_log_l(params, data):
    """
    Negative of the logarithm of the population balance model for use in the MLE minimization
process.
    Inputs:
    data: a list of a single data point containing
    t: Timestep
    L: Fibril length at t
    L0: Initial fibril length
    m0: (Initial) monomer concentration. This has to be replaced with a function or the time-
series data for monomer concentration with time if the monomer concentration is variable.
    params: Vector containing rate constants [k_L, K]

    Output:
    -log(rho(L,t))
    """
    t = data[0]
    L = data[1]
    L0 = data[2]
    M0 = data[3]
    k_L = params[0]
    K = params[1]
    r_g = k_L*M0/(K+M0)
    return np.log(2*np.pi)/2 + np.log(r_g*t)/2 + (1/2/r_g/t)*(L-L0-r_g*t)**2


def cost_func(params, data):
    """
    The cost function to be minimized.
    Inputs:
    params: Vector containing rate constants [k_L, K]
    data: an array containing time-series of fibril length vs. t in the order [t, L, L0, m0]
```

```
    Output:
    running_sum: sum of -log(rho(L,t))
    """
    running_sum = 0
    for i in data:
        running_sum += neg_log_l(params, i)
    return running_sum

opt_res = minimize(fun = cost_func, x0 = [1, 1e-4], method="BFGS", args = (data),
options={'maxiter':5000, 'maxfev':10000, 'fatol':1e-6}) #calling the minimizing algorithm. data is
the timeseries data array in the format described in cost_func.
print(opt_res)
```

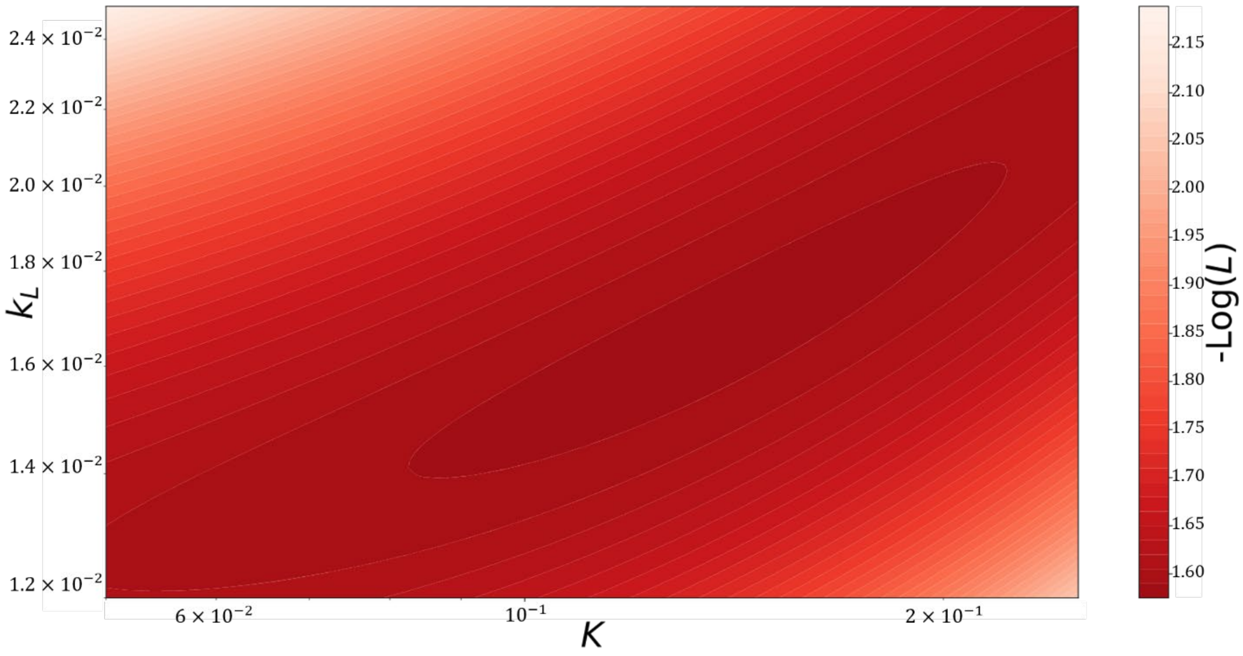

Figure S1. The contour plot of the negative log-likelihood around the minimum.
